# A Latent Profile Analysis of Psychological Functioning During the COVID‐19 Pandemic: Adolescents' Perceived Social Support and Lifestyle Behaviours

**DOI:** 10.1002/ijop.70025

**Published:** 2025-02-27

**Authors:** Giulia Pecora, Fiorenzo Laghi, Roberto Baiocco, Emma Baumgartner, Stefania Sette

**Affiliations:** ^1^ Department of History, Humanities and Society Tor Vergata University Rome Italy; ^2^ Department of Developmental and Social Psychology Sapienza University Rome Italy

**Keywords:** adolescents, latent profile analysis, lifestyle behaviours, perceived social support, psychological functioning

## Abstract

Research highlights notable concerns about adolescents' psychological functioning during the COVID‐19 pandemic, pointing to its association with overall adjustment. Identifying adolescent profiles based on psychological distress risk levels is crucial for developing effective support strategies. This study, conducted with *N* = 579 adolescents (*M*_age_ = 15.97, SD = 1.52), employed a person‐centred approach, using the latent profile analysis (LPA) to identify distinct psychological functioning profiles during the pandemic. Three profiles emerged, including *low‐risk*, *mild‐risk*, and *high‐risk* subgroups, characterised by varying configurations of psychological functioning. The high‐risk subgroup (19.5% of participants) exhibited elevated levels of mental health problems, loneliness, fear of COVID‐19, stress, and negative affect, alongside lower positivity and positive affect. The mild‐risk subgroup (46.8%) demonstrated intermediate scores in the study variables, while the low‐risk subgroup (33.7%) showed the most positive psychological functioning. Differences in perceived social support and lifestyle behaviours were explored among these subgroups. Results showed that the high‐risk subgroup reported lower social support and greater sleep problems, smartphone addiction, and daytime spent on smartphones than the other subgroups. The study underscores the importance of considering the varying risk levels related to adolescents' psychological functioning during emergencies and tailoring interventions to support diverse psychological profiles.

## 1 Introduction

The outbreak of the COVID‐19 pandemic disrupted adolescents' lives on multiple fronts, affecting social interactions, access to school, and daily routines (e.g., Wolf and Schmitz 2024). Relying heavily on peer relationships for emotional support, adolescents became particularly vulnerable to psychological distress due to home confinement and isolation (Magson et al. 2021), experiencing heightened anxiety, depressive symptoms, negative affect, and loneliness (Wolf and Schmitz 2024). Direct and indirect exposure to the virus also contributed to the development of specific illness‐related fears (Soraci et al. 2022), while a lack of positive outlook on life and lower positive affect exacerbated the effects of the pandemic on psychological well‐being (Zdravkovic and Goldstein 2023).

In such a problematic scenario, staying connected with others and adopting healthy habits can represent adaptive methods to mitigate the effects on psychological functioning (Bacaro et al. 2022; Sikorska et al. 2021; Yalçın et al. 2022). While various studies have focused on adolescents' psychological well‐being, there is relatively limited evidence from research employing a person‐centred approach. This approach has the merit of allowing for the identification of distinct subgroups based on different combinations of specific indicators. The present study explored profiles of adolescents based on their vulnerability to psychological distress and examined how these configurations were related to perceived social support and lifestyle behaviours during the pandemic.

### 1.1 Adolescents' Psychological Functioning During the COVID‐19 Pandemic

The pandemic posed remarkable psychological challenges, leading to a surge in loneliness, fear, and stress, especially among adolescents (e.g., Wolf and Schmitz 2024). Adolescence is a critical period of life marked by emotional intensity and instability, involving challenges like identity formation, increased engagement in peer relationships, and initiation of romantic bonds (e.g., Lam et al. 2014). The COVID‐19 pandemic disrupted this phase, with detrimental effects on adolescents' emotional well‐being, with enduring psychological effects. A rise in depressive and anxiety symptoms, as well as a decline in quality of life and psychological well‐being among adolescents, was observed (Wolf and Schmitz 2024). In their longitudinal study, Wang et al. (2023) found that negative affect was positively associated with depressive and anxiety symptoms and feelings of loneliness, especially among adolescents concerned about COVID‐19 infection, lacking parental warmth, and facing disrupted sleep. Loneliness arises when individuals feel dissatisfied with the quantity or quality of their current relationships (Perlman and Peplau 1982). Restrictions to face‐to‐face interactions and social distancing may have interfered with the need for affiliation, particularly relevant during adolescence (Lam et al. 2014), thus leading to a sense of isolation and loneliness.

Amidst the challenges posed by the pandemic, it is crucial to identify factors potentially promoting psychological well‐being. Positivity, an individual's disposition to approach oneself, one's own life, and the future with a positive outlook (Caprara et al. 2012), and affect, a transitory emotional state (Watson et al. 1988), provide important insights into how individuals experience and face challenging events. Enabling individuals to interpret negative experiences more adaptively through cognitive self‐reflection, positivity can promote individuals' psychological well‐being, especially in times of adversity (Caprara et al. 2012). Cirimele et al. (2022) identified three latent profiles of young adults and adults based on individual dispositions and emotional self‐efficacy beliefs during the pandemic. They found that the most resilient profile (18.9%) was positively related to prosocial behaviour and negatively related to interpersonal aggression, depression, and anxiety. Conversely, the most vulnerable profile (22%) displayed the opposite pattern. Similarly, Zdravkovic and Goldstein (2023) individuated two subgroups of adolescents, the “optimists” and the “realists.” Those in the “optimists” profile (24%) demonstrated resilience, characterised by greater positive affect, lower negative affect, and reduced stress during the pandemic. In contrast, adolescents in the “realists” profile (76%) faced more challenges, with increased negative affect and stress, and lower positive affect. Affect and positivity emerge as potential indicators of adolescents' psychological functioning during the pandemic, as they play complementary roles in capturing the complexity of subjective well‐being in adverse situations, encompassing both dispositional and transitory characteristics (Caprara et al. 2012; Watson et al. 1988).

### 1.2 Adolescents' Psychological Functioning, Perceived Social Support, and Lifestyle Behaviours During the Pandemic

The most significant concern for adolescents during the pandemic was the inability to meet or spend time with peers (Magson et al. 2021), leading to feelings of isolation and reduced support. Perceived social support, or the subjective belief regarding the availability and adequacy of care from one's social network (Mai et al. 2021), has been linked to emotional stability and mental health. Yalçın et al. (2022) identified three profiles among undergraduate and graduate students based on varying levels of fear of COVID‐19, psychological symptoms (i.e., depression, anxiety, and stress), mindfulness, and resilience. Social support from family, friends, and a significant other was positively related to the profile of participants (38%) reporting higher mindfulness and resilience and lower psychological symptoms and fear. At the same time, social support was negatively associated with a more vulnerable profile (16%), showing high fear and psychological symptoms, along with low mindfulness and resilience. In another study with a large group of 19‐year‐olds, Essau and de la Torre‐Luque (2021) found that the “high‐symptom” and “emotional dysregulation” profiles experienced increased stress, conflict, and loneliness alongside lower perceived social support. Further investigation is needed to better understand how the diverse sources of social support may be related to adolescents' psychological well‐being in order to identify the most beneficial during times of crisis.

The pandemic also significantly impacted adolescents' lifestyle behaviours, particularly sleep patterns, digital device usage, and physical activity. Disruptions in sleep quality were particularly evident during the pandemic. Overall, alterations in sleep patterns involved extended sleep duration, delayed bedtimes, and poor sleep quality (e.g., Bacaro et al. 2022). In a recent study conducted by Chen et al. (2023), employing latent profile analysis (LPA), college students, particularly those in their second year, experienced elevated rates of sleep disturbances. Additionally, increased depressive symptoms were observed for individuals with mild (37.5%) and high (10.6%) sleep disturbances compared to those presenting low (51.9%) sleep disturbances.

Digital devices became essential during the pandemic for communication, education, entertainment, and information seeking. There is currently a fervent debate in the scientific community about the effects of technology use on adolescents' psychological functioning. Excessive screen time has been linked to poorer mental health (e.g., depression, anxiety, and stress) and lower life satisfaction (Sikorska et al. 2021). At the same time, these effects may vary depending on diverse factors, such as personality traits (e.g., impulsivity, propensity to risk), social support systems, and the nature of online interactions (Busch and McCarthy 2021). Marciano et al. (2022) found that, while certain activities (e.g., one‐to‐one and one‐to‐few communication) effectively alleviated adolescents' feelings of loneliness, excessive digital media usage was linked to mental health problems. Widespread internet access during the pandemic likely led to an overwhelming amount of information about the virus, thus intensifying feelings of worry, anxiety, and fear. It should be noted that playing online games during the pandemic was also positively related to mental health problems. According to Pallavicini et al. (2022), while gaming initially relieved stress, anxiety, and depression, it became maladaptive for individuals at higher risk, such as problematic gamers and male youths.

Notably, physical activity declined and sedentary behaviour increased during the pandemic, negatively affecting physical health. As Rossi et al. (2021) noted, preventive restrictions imposed in many countries reduced adolescents' opportunities and motivation for physical activity, leading to poorer outcomes for their physical health.

Overall, existing research underlines concerns about adolescents' psychological functioning during the COVID‐19 pandemic. The studies conducted so far have shown that adopting a person‐centred approach can help better understand the characteristics of adolescents who were most affected by the consequences of the pandemic. Further investigation is warranted to explore how adolescents' perceived social support and lifestyle behaviours interplay with different levels of psychological functioning vulnerability.

### 1.3 The Current Study

The present study seeks to extend prior literature by adopting a person‐centred approach. We conducted an LPA, grouping adolescents based on their vulnerability in psychological functioning, defined by mental health, stress, loneliness, and fear of COVID‐19, as well as positivity and positive/negative affect (e.g., Soraci et al. 2022; Wang et al. 2023; Zdravkovic and Goldstein 2023). Subsequently, we investigated how the individuated subgroups differed regarding their perceived social support and lifestyle behaviours. We expected that more vulnerable subgroups would present lower means in perceived social support (e.g., Yalçın et al. 2022) and sleep quality (e.g., Chen et al. 2023), as well as higher screen time (e.g., Sikorska et al. 2021) and less engagement in physical activities (e.g., Rossi et al. 2021) compared to less vulnerable subgroups.

## 2 Method

### 2.1 Participants

The study included *N* = 579 adolescents (49.5% females) aged 13 to 21 (*M*_age_ = 15.97, SD = 1.52). Participants were enrolled in secondary schools in a large metropolitan area in Italy. To be included in the study, adolescents had to attend a secondary school and be proficient in the Italian language. Most participants (97.57%) were native‐born Italians, with the remaining respondents having a migratory background (1.39% from East Europe, 0.52% from America, 0.35% from Asia, and 0.17% from Africa). For two participants, data were missing. The prevailing socio‐economic status (SES) of the participants was middle class (77.6%), followed by upper‐middle class (19%).

### 2.2 Procedure

The data collected in this study are part of a broader project that explores the mental health and lifestyle behaviours of adolescents in secondary schools during the COVID‐19 pandemic (Pecora et al. 2024). The Research Ethics Board approved this research project at the Department of Developmental and Social Psychology, Sapienza University of Rome. After obtaining informed consent from schools, parents, and participants, we asked students to complete an online questionnaire. The measures encompassed psychological functioning (i.e., mental health problems, loneliness, fear of COVID‐19, stress, positivity, positive and negative affect), perceived social support, and lifestyle behaviours (i.e., sleep problems, smartphone and video game addictions, physical activity). The data collection was carried out between February and May 2021. This period followed the initial waves of the COVID‐19 pandemic and coincided with significant fluctuations in infection rates, vaccination deployment, and changes in public health guidelines. Furthermore, schools were employing new protocols, with intermittent closures and shifts between fully remote, fully in‐person, and hybrid learning. All these changes likely had an influence on adolescents' psychological functioning. The survey was conducted using the Qualtrics survey tool.

### 2.3 Measures

#### 2.3.1 Psychological Functioning

##### 2.3.1.1 Mental Health Problems

The 12‐item General Health Questionnaire (GHQ‐12; Goldberg et al. 1997) was used to assess adolescents' risk of developing psychological problems, encompassing the domains of anxiety, depression, somatic symptoms, and social withdrawal. Participants rated each item (e.g., *Have you recently felt unhappy and depressed?*) on a 4‐point scale ranging from 0 (*significantly less than usual*) to 3 (*more than usual*). Higher scores indicate greater difficulties in psychological well‐being (*α* = 0.86).

##### 2.3.1.2 Stress

Participants completed the stress subscale of the Depression Anxiety Stress Scale—21 (DASS‐21; Lovibond and Lovibond 1995). This measure consisted of seven items (e.g., *I tended to overreact to situations*) to be rated on a 4‐point scale ranging from 1 (*doesn't apply to me at all*) to 4 (*applies a lot or most of the time to me*; *α* = 0.89).

##### 2.3.1.3 Loneliness

We administered a shortened version of the 20‐item *UCLA Loneliness Scale* (Russell et al. 1978), assessing subjective feelings of loneliness and social isolation. We selected four items (e.g., *I feel isolated from others*) to alleviate participants' burden associated with questionnaire completion. Participants rated each item on a 4‐point scale, ranging from 0 (*I never feel this way*) to 3 (*I often feel this way*; *α* = 0.85).

##### 2.3.1.4 Fear of COVID‐19

Participants completed the Fear of COVID‐19 Scale (FCV‐19S; Ahorsu et al. 2022), assessing the extent of fear experienced by individuals in response to COVID‐19. A one‐dimensional structure characterised the instrument and consisted of seven items (e.g., *It makes me uncomfortable to think about coronavirus‐19*) rated on a 5‐point scale, ranging from 1 (*strongly disagree*) to 5 (*strongly agree*; *α* = 0.86).

##### 2.3.1.5 Positive and Negative Affect

We evaluated affect using the Positive and Negative Affect Schedule (PANAS; Watson et al. 1988). This scale consists of 20 items, with 10 focusing on positive affect (e.g., *excited*) and 10 on negative affect (e.g., *nervous*). Participants indicated the extent to which they experienced a specific affect on a 5‐point scale ranging from 1 (*very slightly or not at all*) to 5 (*extremely*; positive affect: *α* = 0.88; negative affect: *α* = 0.87).

##### 2.3.1.6 Positivity

We used the Positivity scale (Caprara et al. 2012) to measure the inclination to appraise one's life and experiences with a positive outlook. This scale comprised eight items (e.g., *I have great faith in the future*) covering self‐esteem, optimism, and life satisfaction. Participants rated each item on a 5‐point scale ranging from 1 (*strongly disagree*) to 5 (*strongly agree*; *α* = 0.78).

#### 2.3.2 Perceived Social Support

We asked participants to provide insights into the level of social support they perceived within their relationships. Initially, referencing the last two weeks, we asked, “How many people close to you can you really count on for help and support in times of trouble?”. Participants responded on a 4‐point scale, from 1 (*0 people*) to 5 (*more than 10 people*). Subsequently, we asked participants to indicate how often they turned to specific individuals for support in times of difficulty in the preceding two weeks. The individuals included parents, siblings, relatives, friends, romantic partners, and teachers. Participants responded for each person on a 4‐point scale ranging from 1 (*never*) to 4 (*always*).

#### 2.3.3 Lifestyle Behaviours

##### 2.3.3.1 Sleep Problems

We administered the Sleep–Wake Problems Behaviour Scale (SWPBS), which was extracted from the School Sleep Habits Survey (Wolfson et al. 2003). This instrument allowed us to evaluate sleep‐related challenges, including waking up in the morning and awakening at night. While the original scale comprises 15 items, we opted for a shortened version with six items to minimise the testing duration and prevent participants' fatigue. Respondents rated each item (e.g., *In the last two weeks, how often have you had an extremely hard time falling asleep?*) on a 5‐point scale ranging from 1 (*never*) to 5 (*always*; *α* = 0.71).

##### 2.3.3.2 Smartphone Addiction

We examined participants' smartphone addiction using four items from the Shorter PROMIS Questionnaire (SPQ; Tafà and Baiocco 2009). This instrument assesses individuals' smartphone misuse through items (e.g., *I often find myself using my phone much more than I would like*) on a 6‐point scale ranging from 1 (*absolutely false for me*) to 6 (*absolutely true for me*; *α* = 0.62). Adolescents also indicated the *daytime spent with smartphones* (number of hours per day), with responses ranging from 0 to 24 h.

##### 2.3.3.3 Video Game Addiction

We assessed adolescents' video game addiction through 4 items adjusted from the SPQ (Tafà and Baiocco 2009). The measure evaluates an individual's gaming addiction via items (e.g., *When I am with my family, friends, or other people, I tend to conceal the excessive use of video games*) on a 6‐point scale ranging from 1 (*absolutely false for me*) to 6 (*absolutely true for me*; *α* = 0.74).

##### 2.3.3.4 Physical Activity

We asked adolescents to indicate whether they engaged in physical activities and to specify the number of hours spent doing a sport each week. Participants selected the appropriate number from a list ranging from 0 to 40 h (per week).

### 2.4 Socio‐Demographic Information

Adolescents provided details about their age, sex assigned at birth (0 = *male*, 1 = *female*, 2 = *other*), place of birth, and the migratory background of both themselves and their parents' (indicating where they and their parents were born). The SES was investigated by asking “How would you define your SES condition?”. Participants responded on a 5‐point scale ranging from 1 (*extremely low*) to 5 (*extremely high*).

### 2.5 Plan of the Analysis

We calculated descriptive statistics and Pearson's correlations among the study variables. Then, we performed an LPA, including mental health problems, loneliness, fear of COVID‐19, positivity, stress, and positive and negative affect. We estimated models with one‐ to sixth‐class solutions using the number of starting values of 500 and the number of optimisation steps of 100, without incorporating any other model specifications. Each variable was standardised in the LPA for better interpretability of each class solution. We employed multiple fit indices, model parsimony, and theoretical considerations to select the optimal class solution. Regarding model fit indices for the best class solution, we took into account (1) lower log‐likelihood, Akaike Information Criterion (AIC), Bayesian Information Criterion (BIC), and Sample‐Adjusted BIC, (2) entropy closer to 1, and (3) significant Lo‐Mendell Rubin (LMR) and Bootstrap Likelihood Ratio Test (BLRT; Ferguson et al. 2020). Additionally, each identified subgroup needed to constitute at least 5% of the sample for the chosen class solution (Speece 1994). We utilised the Maximum Likelihood Estimator with Robust Standard Errors (MLR) to estimate models.

We conducted a Chi‐square analysis to examine differences in sex assigned at birth within each identified subgroup. Then, to explore potential differences among the identified subgroups on variables within the LPA and additional factors (i.e., perceived social support, sleep problems, smartphone and video game addictions, daytime spent with a smartphone, and physical activity), we conducted a series of Analyses of Variance (ANOVAs) with Bonferroni post hoc comparisons. The analyses were performed using SPSS 27 and MPlus 8.11.

## 3 Results

### 3.1 Latent Profile Analysis (LPA): Psychological Functioning

**FIGURE 1:**
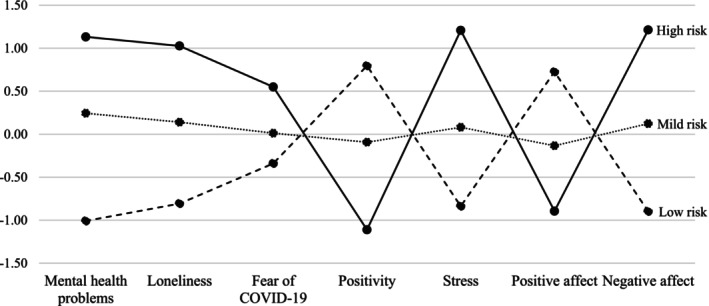
Results of latent profile analysis (LPA) with three‐class solution. For each variable, we represented standardised means.

Correlation analyses revealed that the study variables were associated in the expected directions (see Table S1). We estimated six‐class solutions on the entire sample (see Table S2). Although the highest log‐likelihood (most close to zero) and lowest AIC, BIC, and SABIC indicated that the 4‐5‐6 class model solutions fit the data better than others, we rejected these models due to a lack of theoretical interpretation. Based on these results, the highest entropy value, the highest log‐likelihood, and the lowest AIC, BIC, and SABIC compared to models 1 and 2, and theory, we selected Model 3, which comprised three distinct subgroups. These were designated as *low‐risk* (*n* = 195, 33.7%), *mild‐risk* (*n* = 271, 46.8%), and *high‐risk* (*n* = 113, 19.5%). As presented in Figure 1, different profiles emerged for each subgroup. ANOVAs with Bonferroni post hoc comparisons indicated significant differences in psychological functioning across the three subgroups. Specifically, the *high‐risk* subgroup reported significantly higher scores on mental health problems, *F*(2,566) = 495.13, *p* < 0.001, *η* ^2^_*p*_ = 0.64, loneliness, *F*(2,558) = 235.32, *p* < 0.001, *η* ^2^_*p*_ = 0.46, fear of COVID‐19, *F*(2,577) = 30.23, *p* < 0.001, *η* ^2^_*p*_ = 0.09, stress, *F*(2,556) = 314.04, *p* < 0.001, *η* ^2^_*p*_ = 0.53, and negative affect, *F*(2,553) = 384.95, *p* < 0.001, *η* ^2^_*p*_ = 0.58, than the mild and low‐risk subgroups. In turn, the *mild‐risk* subgroup presented higher scores on the same variables than the low‐risk subgroup. An opposite pattern emerged for positivity and positive affect. Indeed, the *low‐risk* subgroup reported significantly higher scores in positivity, *F*(2,560) = 260.25, *p* < 0.001, *η* ^2^_*p*_ = 0.48, and positive affect, *F*(2,552) = 160.50, *p* < 0.001, *η* ^2^_*p*_ = 0.37, than the mild and high‐risk subgroups. The mild‐risk subgroup reported higher scores on both variables than the high‐risk subgroup.

### 3.2 Differences Among LPA Subgroups in Perceived Social Support, Lifestyle Behaviours, and Socio‐Demographic Variables

To investigate the differences between each psychological functioning subgroup, we conducted a series of ANOVAs on the remaining study variables (Table 1, ^1^). Results revealed that the high‐risk subgroup reported lower means than the low‐risk subgroup for perceived social support, indicating fewer people on whom they felt they could count. Similar findings emerged for perceived social support from parents, relatives, and teachers. Additionally, the high‐risk subgroup reported perceiving lower social support from friends than both the mild and low‐risk subgroups. The high‐risk subgroup exhibited higher scores than the other two subgroups on sleep problems, smartphone addiction, and daytime spent using a smartphone. The mild‐risk subgroup presented lower means than the low‐risk subgroup on social support in terms of the number of people on whom they perceived they could count. Similar findings emerged for perceived social support from parents, relatives, and teachers. The mild‐risk subgroup reported higher means than the low‐risk subgroup on sleep problems, smartphone addiction, and daytime spent with a smartphone. No significant differences emerged among the subgroups regarding perceived social support from siblings and the romantic partner, video game addiction, and the number of hours spent in physical activities.

**TABLE 1:** Characteristics of the three subgroups from latent profile analysis.

Variable	High risk	Mild risk	Low risk	*F*	*η* ^2^_*p*_
Social support	2.95_b_	3.09_b_	3.52_a_	27.30	0.09
Support from parents	2.26_b_	2.45_b_	2.72_a_	11.37	0.04
Support from siblings	1.66_a_	1.81_a_	1.91_a_	2.40	0.01
Support from relatives	1.35_b_	1.47_b_	1.82_a_	21.00	0.07
Support from friends	2.59_b_	2.86_a_	2.91_a_	5.55	0.02
Support from a romantic partner	1.75_a_	1.74_a_	1.73_a_	0.02	0.00
Support from teachers	1.22_b_	1.35_b_	1.52_a_	10.32	0.03
Sleep problems	3.54_a_	3.03_b_	2.42_c_	104.93	0.27
Smartphone addiction	3.90_a_	3.15_b_	2.65_c_	56.36	0.17
Video game addiction	2.00_a_	1.92_a_	2.02_a_	0.49	0.00
Daytime spent with smartphone	6.80_a_	5.77_b_	5.08_c_	13.12	0.05
Physical activity (number of hours)	5.50_a_	6.37_a_	6.67_a_	2.52	0.01

*Note:* Degrees of freedom were df = 3.344 for all variables; *η* ^2^_*p*_ indicates partial eta‐squared. Means with different subscripts in the same row differ at *p* < 0.05 (Bonferroni *p* value for multiple comparisons).

Age differences emerged among the latent profiles, *F*(2,577) = 8.76, *p* < 0.001, *η* ^2^_*p*_ = 0.03, showing a lower mean age for the low‐risk subgroup (*M* = 15.61) compared to the mild‐risk (*M* = 16.18) and the high‐risk (*M* = 16.12) subgroups. Moreover, Chi‐square analysis revealed significant sex assigned at birth differences among the subgroups (*χ* ^2^(2) = 72.032, *p* < 0.001). Specifically, more females than expected were found in the high‐risk subgroup (*n* = 85, 75.9%, vs. *n* = 27 males, 24.1%), whereas more males than expected were found in the low‐risk subgroup (*n* = 141, 72.7%, vs. *n* = 53 females, 27.3%). No other differences between the observed and the expected frequencies were found for the mild‐risk subgroup (*n* = 123 males, 45.5%, vs. *n* = 147 females, 55.4%) in the sex assigned at birth.

## 4 Discussion

The LPA revealed three distinct subgroups of adolescents based on their psychological functioning during the pandemic: low‐risk, mild‐risk, and high‐risk. These profiles varied in mental health problems, loneliness, fear of COVID‐19, stress, positivity, and positive and negative affect. Delving deeper into our results, the high‐risk subgroup exhibited greater vulnerability across various dimensions, with higher levels of mental health problems, loneliness, fear of COVID‐19, stress, and negative affect, alongside lower levels of positivity and positive affect. In contrast, the low‐risk subgroup reported more favourable psychological functioning, characterised by lower levels of mental health problems, loneliness, fear of COVID‐19, stress, and negative affect, as well as higher levels of positivity and positive affect. Finally, the mild‐risk subgroup fell between these two subgroups, showing intermediate scores on all dimensions of psychological functioning. These findings align with previous research using the LPA, emphasising the diversity of responses during challenging times (Cirimele et al. 2022; Yalcin et al. 2021). The person‐centred approach was crucial in taking into account the heterogeneity of adolescent responses that variable‐oriented approaches might overlook. Notably, the low‐risk subgroup showed a heightened capacity for adaptation, with higher positivity and positive affect playing a crucial role in protecting against mental health issues (e.g., Caprara et al. 2012). Conversely, lower resilience may contribute to greater vulnerability for adolescents falling within the high‐risk profile. These findings are in line with prior studies (e.g., Cirimele et al. 2022) showing that individuals with higher vulnerability and lower self‐efficacy beliefs presented greater maladaptive outcomes, including interpersonal aggression, depression, and anxiety during the pandemic.

Perceived social support played a crucial role in differentiating the psychological functioning subgroups. The high‐risk subgroup reported lower perceived social support than the low‐risk subgroup across multiple domains, including support from parents, relatives, friends, and teachers. These findings align with research highlighting social support as a protective factor against stress, negative affect, and mental health issues during the pandemic (e.g., Yalçın et al. 2022). They also underscore the importance of social support in adolescence, when the need for affiliation with peers becomes preeminent (e.g., Lam et al. 2014). Restrictions on social interactions may have disrupted this need. These results may contribute to a better understanding of the interplay between different levels of vulnerability in psychological functioning and perceived social support during the COVID‐19 pandemic.

The study also highlights the associations between adolescents' psychological functioning and lifestyle behaviours. The high‐risk subgroup reported more sleep problems, smartphone addiction, and increased smartphone usage during the day, while the low‐risk subgroup, characterised by more favourable psychological functioning, demonstrated healthier habits, including better sleep quality, lower smartphone addiction, and less time spent with the smartphone during the day. For the mild‐risk subgroup, exhibiting intermediate levels of psychological functioning, lifestyle behaviours fell in between those of the other two subgroups. These results are consistent with prior studies showing that youth experiencing psychological issues, including depression symptoms, stress, and anxiety, were more prone to sleep disturbances (Chen et al. 2023) and excessive digital device use (Sikorska et al. 2021). The pervasive sense of uncertainty, social isolation, and disruptions in daily routines during the pandemic might have intensified emotional distress, exacerbating sleep disturbances among vulnerable individuals (Bacaro et al. 2022). Moreover, adolescents may rely heavily on their smartphones during times of heightened emotional turmoil. On the one hand, digital devices can provide an immediate escape from overwhelming emotions (Sikorska et al. 2021) and serve as an avoidant strategy (Busch and McCarthy 2021), likely hindering emotional processing and contributing to enhanced psychological distress. On the other hand, during social isolation, smartphones represented a vital tool for social support, facilitating connections with friends and significant others (Marciano et al. 2022), eventually reinforcing the affiliation need, which is so important during this developmental stage (Lam et al. 2014). This may be particularly true for high‐risk adolescents, who are more vulnerable to psychological distress exacerbated by the pandemic. However, as our findings are based on cross‐sectional data, the directionality of the interplay between digital device use and psychological outcomes remains unclear. To note, as this study took place during a blended learning period, increased screen time for academic purposes should be considered. However, our focus on smartphone addiction specifically examined its link to negative emotional states, such as worry and anger.

We found no significant differences in gaming addiction across the subgroups. While excessive gaming is often associated with negative outcomes (Marciano et al. 2022), our results suggest that playing video games may not have contributed to variations in risk profiles during the pandemic. Pallavicini et al. (2022) noted that gaming served as a coping strategy in the initial phases of the pandemic and became maladaptive later for problematic gamers and male youths. Importantly, gaming addiction levels in our sample were relatively low, reflecting a non‐clinical trend. Moreover, the impact of gaming may depend on its nature. For instance, multiplayer online games that foster social interaction and collaboration may have distinct effects compared to solitary, highly competitive games. Future research should explore these dynamics further.

Older adolescents and females were overrepresented in the high‐risk subgroup, suggesting their increased vulnerability. Females tend to rely on social relationships for support more than males when facing significant life stressors (e.g., Magson et al. 2021). Consequently, restrictions on social interactions during this period may have affected an essential coping strategy. Similarly, older adolescents often seek support from peers rather than from parents and family members (Lam et al. 2014). Therefore, social distancing might have been more challenging for them than for younger adolescents.

### 4.1 Strengths, Limitations, and Future Directions

The strengths of this study include its person‐centred approach, acknowledging the heterogeneity of adolescents' experiences during the COVID‐19 pandemic. This method provided a comprehensive understanding of how different dimensions of psychological functioning (e.g., fear of COVID‐19, positivity, stress) interacted to define risk profiles. This is crucial for developing intervention programmes that address the specific needs of different adolescent subgroups, which may be overlooked using variable‐centred approaches. Furthermore, the study employed a large set of measures encompassing psychological functioning, perceived social support, and lifestyle behaviours.

However, some limitations should be noted. The study adopted a cross‐sectional design, limiting the ability to establish causal relations. Future research employing longitudinal designs could offer deeper insights into these associations over time. Moreover, self‐report measures may introduce the potential for bias and inaccuracy, which future research could mitigate through multi‐method approaches, including observation, interviews, and questionnaires administered to others (e.g., parents). Finally, although a minimum of about 500 participants should be enough for accuracy in the identification of the number of latent profiles (Nylund et al. 2007), the robustness and reliability of our findings could be increased by including larger samples in future research.

Overall, this study provides valuable insights into the diversity of adolescents' responses to the pandemic and underscores the importance of social support and lifestyle behaviours in relation to their psychological functioning. By recognising the diverse needs of adolescents, policymakers, educators, and mental health professionals can help foster adaptive coping strategies and promote social support in the face of ongoing global challenges. Since positivity played an important protective role for adolescents within the low‐risk subgroup in our sample, interventions could strategically integrate this relevant factor.

## Ethics Statement

The current study was approved by the Ethics Committee of the Department of Developmental and Social Psychology, Sapienza University, Rome. The study was performed in accordance with the ethical standards as set forth in the 1964 Declaration of Helsinki and its later amendments.

## Consent

All participants were informed of the purpose of the study and signed informed consent.

## Conflicts of Interest

The authors declare no conflicts of interest.

## Supporting information


Data S1.

## Data Availability

The dataset for this original article is available at this link https://osf.io/2sed9/?view_only=eb910de0b63b435b97a998096e50eb97.

## Endnotes

^1^
As requested by an anonymous reviewer, we also ran the three‐step LPA approach (Vermunt 2010). We added 12 covariates and included them as predictors in the best conditional LPA model (with the Mplus command auxiliary = R3STEP). However, after the individuation of the latent profiles (*N* = 579), listwise deletion was applied to the auxiliary variables in the analysis (number of observations used *n* = 346), thereby, excluding a relevant portion of the total sample (*n* = 233). For more details of these results, see the Supporting Information.

## References

[ijop70025-bib-0001] Ahorsu, D. K. , C. Y. Lin , V. Imani , M. Saffari , M. D. Griffiths , and A. H. Pakpour . 2022. “The Fear of COVID‐19 Scale: Development and Initial Validation.” International Journal of Mental Health and Addiction 20, no. 3: 1537–1545. 10.1007/s11469-020-00270-8. 32226353 PMC7100496

[ijop70025-bib-0002] Bacaro, V. , D. Meneo , S. Curati , et al. 2022. “The Impact of COVID‐19 on Italian Adolescents' Sleep and Its Association With Psychological Factors.” Journal of Sleep Research 31: e13689. 10.1111/jsr.13689. 35830968 PMC9349647

[ijop70025-bib-0003] Busch, P. A. , and S. McCarthy . 2021. “Antecedents and Consequences of Problematic Smartphone Use: A Systematic Literature Review of an Emerging Research Area.” Computers in Human Behavior 114: 106414. 10.1016/j.chb.2020.106414.

[ijop70025-bib-0004] Caprara, G. V. , G. Alessandri , N. Eisenberg , et al. 2012. “The Positivity Scale.” Psychological Assessment 24, no. 3: 701–712. 10.1037/a0026681. 22250591

[ijop70025-bib-0005] Chen, C. , Z. He , B. Xu , J. Shao , and D. Wang . 2023. “A Latent Profile Analysis of Sleep Disturbance in Relation to Mental Health Among College Students in China.” Frontiers in Public Health 11: 1107692. 10.3389/fpubh.2023.1107692. 37325305 PMC10266341

[ijop70025-bib-0006] Cirimele, F. , C. Pastorelli , A. Favini , et al. 2022. “Facing the Pandemic in Italy: Personality Profiles and Their Associations With Adaptive and Maladaptive Outcomes.” Frontiers in Psychology 13: 805740. 10.3389/fpsyg.2022.805740. 35282221 PMC8908009

[ijop70025-bib-0007] Essau, C. A. , and A. de la Torre‐Luque . 2021. “Adolescent Psychopathological Profiles and the Outcome of the COVID‐19 Pandemic: Longitudinal Findings From the UK Millennium Cohort Study.” Progress in Neuropsychopharmacology and Biological Psychiatry 110: 110330. 10.1016/j.pnpbp.2021.110330. PMC8569420 33887389

[ijop70025-bib-0008] Ferguson, S. L. , G. Moore , E. W , and D. M. Hull . 2020. “Finding Latent Groups in Observed Data: A Primer on Latent Profile Analysis in Mplus for Applied Researchers.” International Journal of Behavioral Development 44, no. 5: 458–468. 10.1177/0165025419881721.

[ijop70025-bib-0009] Goldberg, D. P. , R. Gater , N. Sartorius , et al. 1997. “The Validity of Two Versions of the GHQ in the WHO Study of Mental Illness in General Health Care.” Psychological Medicine 27, no. 1: 191–197. 10.1017/s0033291796004242. 9122299

[ijop70025-bib-0010] Lam, C. B. , S. M. McHale , and A. C. Crouter . 2014. “Time With Peers From Middle Childhood to Late Adolescence: Developmental Course and Adjustment Correlates.” Child Development 85, no. 4: 1677–1693. 10.1111/cdev.12235. 24673293 PMC4107039

[ijop70025-bib-0011] Lovibond, P. F. , and S. H. Lovibond . 1995. “The Structure of Negative Emotional States: Comparison of the Depression Anxiety Stress Scales (DASS) With the Beck Depression and Anxiety Inventories.” Behaviour Research and Therapy 33, no. 3: 335–343. 10.1016/0005-7967(94)00075-U. 7726811

[ijop70025-bib-0012] Magson, N. R. , J. Y. A. Freeman , R. M. Rapee , C. E. Richardson , E. L. Oar , and J. Fardouly . 2021. “Risk and Protective Factors for Prospective Changes in Adolescent Mental Health During the COVID‐19 Pandemic.” Journal of Youth and Adolescence 50, no. 1: 44–57. 10.1007/s10964-020-01332-9. 33108542 PMC7590912

[ijop70025-bib-0013] Mai, Y. , Y. J. Wu , and Y. Huang . 2021. “What Type of Social Support Is Important for Student Resilience During COVID‐19? A Latent Profile Analysis.” Frontiers in Psychology 12: 646145. 10.3389/fpsyg.2021.646145. 34239476 PMC8258314

[ijop70025-bib-0014] Marciano, L. , M. Ostroumova , P. J. Schulz , and A.‐L. Camerini . 2022. “Digital Media Use and Adolescents' Mental Health During the Covid‐19 Pandemic: A Systematic Review and Meta‐Analysis.” Frontiers in Public Health 9: 793868. 10.3389/fpubh.2021.793868. 35186872 PMC8848548

[ijop70025-bib-0015] Nylund, K. L. , T. Asparouhov , and B. O. Muthén . 2007. “Deciding on the Number of Classes in Latent Class Analysis and Growth Mixture Modeling: A Monte Carlo Simulation Study.” Structural Equation Modeling: A Multidisciplinary Journal 14, no. 4: 535–569. 10.1080/10705510701575396.

[ijop70025-bib-0016] Pallavicini, F. , A. Pepe , and F. Mantovani . 2022. “The Effects of Playing Video Games on Stress, Anxiety, Depression, Loneliness, and Gaming Disorder During the Early Stages of the COVID‐19 Pandemic: PRISMA Systematic Review.” Cyberpsychology, Behavior, and Social Networking 25, no. 6: 334–354. 10.1089/cyber.2021.0252. 35639118

[ijop70025-bib-0031] Pecora, G. , F. Laghi , E. Baumgartner , A. Di Norcia , and S. Sette . 2024. “The Role of Loneliness and Positivity on Adolescents' Mental Health and Sleep Quality During the COVID‐19 Pandemic". Current Psychology 43: 23352–23365. 10.1007/s12144-024-05805-z.

[ijop70025-bib-0017] Perlman, D. , and L. Peplau . 1982. “Perspectives on Loneliness.” In Loneliness: A Sourcebook of Current Theory, Research and Therapy, edited by L. Peplau and D. Perlman , 1–18. John Wiley and Sons.

[ijop70025-bib-0018] Rossi, L. , N. Behme , and C. Breuer . 2021. “Physical Activity of Children and Adolescents During the COVID‐19 Pandemic – A Scoping Review.” International Journal of Environmental Research and Public Health 18: 11440. 10.3390/ijerph182111440. 34769956 PMC8583307

[ijop70025-bib-0019] Russell, D. , L. A. Peplau , and M. L. Ferguson . 1978. “Developing a Measure of Loneliness.” Journal of Personality Assessment 42, no. 3: 290–294. 10.1207/s15327752jpa4203_11. 660402

[ijop70025-bib-0020] Sikorska, M. I. , N. Lipp , P. Wróbel , and M. Wyra . 2021. “Adolescent Mental Health and Activities in the Period of Social Isolation Caused by the COVID‐19 Pandemic.” Advances in Psychiatry and Neurology 30, no. 2: 79–95. 10.5114/ppn.2021.108472. 37082432 PMC9881619

[ijop70025-bib-0021] Soraci, P. , A. Ferrari , F. A. Abbiati , et al. 2022. “Validation and Psychometric Evaluation of the Italian Version of the Fear of COVID‐19 Scale.” International Journal of Mental Health and Addiction 20: 1913–1922. 10.1007/s11469-020-00277-1. 32372892 PMC7198091

[ijop70025-bib-0022] Speece, D. L. 1994. “Cluster Analysis in Perspective.” Exceptionality 5, no. 1: 31–44. 10.1207/s15327035ex0501_3.

[ijop70025-bib-0023] Tafà, M. , and R. Baiocco . 2009. “Addictive Behavior and Family Functioning During Adolescence.” American Journal of Family Therapy 37: 388–395. 10.1080/01926180902754745.

[ijop70025-bib-0024] Vermunt, J. K. 2010. “Latent Class Modeling With Covariates: Two Improved Three‐Step Approaches.” Political Analysis 18: 450–469. 10.1093/pan/mpq025.

[ijop70025-bib-0025] Wang, M.‐T. , D. A. Henry , C. L. Scanlon , J. Del Toro , and S. E. Voltin . 2023. “Adolescent Psychosocial Adjustment During COVID‐19: An Intensive Longitudinal Study.” Journal of Clinical Child & Adolescent Psychology 52, no. 5: 633–648. 10.1080/15374416.2021.2007487. 35007446

[ijop70025-bib-0026] Watson, D. , L. A. Clark , and A. Tellegen . 1988. “Development and Validation of Brief Measures of Positive and Negative Affect: The PANAS Scales.” Journal of Personality and Social Psychology 54, no. 6: 1063–1070. 10.1037/0022-3514.54.6.1063. 3397865

[ijop70025-bib-0027] Wolf, K. , and J. Schmitz . 2024. “Scoping Review: Longitudinal Effects of the COVID‐19 Pandemic on Child and Adolescent Mental Health.” European Child & Adolescent Psychiatry 33: 1257–1312. 10.1007/s00787-023-02206-8. 37081139 PMC10119016

[ijop70025-bib-0028] Wolfson, A. R. , M. A. Carskadon , C. Acebo , et al. 2003. “Evidence for the Validity of a Sleep Habits Survey for Adolescents.” Sleep 26, no. 2: 213–216. 10.1093/sleep/26.2.213. 12683482

[ijop70025-bib-0029] Yalçın, İ. , N. Can , Ö. Mançe Çalışır , S. Yalçın , and B. Çolak . 2022. “Latent Profile Analysis of COVID‐19 Fear, Depression, Anxiety, Stress, Mindfulness, and Resilience.” Current Psychology 41: 459–469. 10.1007/s12144-021-01667-x. 33821112 PMC8012016

[ijop70025-bib-0030] Zdravkovic, A. , and A. L. Goldstein . 2023. “Optimists and Realists: A Latent Class Analysis of Students Graduating From High School During COVID‐19 and Impacts on Affect and Well‐Being.” International Journal of Environmental Research and Public Health 20: 2120. 10.3390/ijerph20032120. 36767487 PMC9915344

