# Supplementary material for: A Latent Profile Analysis of Psychological Functioning During the COVID‐19 Pandemic: Adolescents' Perceived Social Support and Lifestyle Behaviours
Source: Int J Psychol. 2025 Feb 27;60(2):e70025. doi: 10.1002/ijop.70025 (PMC11867979; doi:10.1002/ijop.70025)
Supplement: Supplementary file 1 — Data S1. [file IJOP-60-e70025-s001.docx]

**A Latent Profile Analysis of Psychological Functioning during the COVID-19 Pandemic: Adolescents’ Perceived Social Support and Lifestyle Behaviours**

Supplementary Information

**Table S1**

*Descriptive Statistics and Zero-Order Correlations among the Study Variables*

|  | *Mean* | *SD* | 1. | 2. | 3. | 4. | 5. | 6. | 7. | 8. | 9. | 10. | 11. | 12. | 13. | 14. | 15. | 16. | 17. | 18. | 19. | 20. | 21. |
| --- | --- | --- | --- | --- | --- | --- | --- | --- | --- | --- | --- | --- | --- | --- | --- | --- | --- | --- | --- | --- | --- | --- | --- |
| 1. Mental health problems | 1.85 | 0.55 | – |  |  |  |  |  |  |  |  |  |  |  |  |  |  |  |  |  |  |  |  |
| 2. Loneliness | 2.52 | 0.83 | .53** | – |  |  |  |  |  |  |  |  |  |  |  |  |  |  |  |  |  |  |  |
| 3. Fear of COVID-19 | 2.27 | 0.76 | .23** | .20** | – |  |  |  |  |  |  |  |  |  |  |  |  |  |  |  |  |  |  |
| 4. Positivity | 3.33 | 0.75 | -.57** | -.54** | -.09* | – |  |  |  |  |  |  |  |  |  |  |  |  |  |  |  |  |  |
| 5. Stress | 1.46 | 0.76 | .62** | .44** | .34** | -.42** | – |  |  |  |  |  |  |  |  |  |  |  |  |  |  |  |  |
| 6. Positive affect | 2.71 | 0.78 | -.56** | -.46** | -.10* | .62** | -.32** | – |  |  |  |  |  |  |  |  |  |  |  |  |  |  |  |
| 7. Negative affect | 2.65 | 0.86 | .63** | .50** | .36** | -.47** | .78** | -.31** | – |  |  |  |  |  |  |  |  |  |  |  |  |  |  |
| 8. Social support | 3.20 | 0.77 | -.25** | -.35** | -.01 | .37** | -.16** | .27** | -.17** | – |  |  |  |  |  |  |  |  |  |  |  |  |  |
| 9. Support from parents | 2.50 | 0.87 | -.15** | -.22** | .10* | .36** | -.10* | .21** | -.08 | .28** | – |  |  |  |  |  |  |  |  |  |  |  |  |
| 10. Support from siblings | 1.81 | 0.94 | -.12** | -.15** | .09* | .20** | -.06 | .16** | -.02 | .13** | .30** | – |  |  |  |  |  |  |  |  |  |  |  |
| 11. Support from relatives | 1.56 | 0.72 | -.25** | -.25** | .01 | .37** | -.18** | 23** | -.17** | .31** | .45** | .24** | – |  |  |  |  |  |  |  |  |  |  |
| 12. Support from friends | 2.82 | 0.84 | -.12** | -.24** | -.02 | .24** | .02 | .19** | -.02 | .30** | .15** | .13** | .16** | – |  |  |  |  |  |  |  |  |  |
| 13. Support from romantic partner | 1.74 | 1.14 | .02 | -.06 | .16** | .16** | .17** | .10* | .13** | -.01 | .08 | .07 | .02 | .08* | – |  |  |  |  |  |  |  |  |
| 14. Support from teachers | 1.38 | 0.58 | -.19** | -.16** | .03 | .30** | -.14** | .25** | -.13** | .22** | .31** | .09* | .32** | .22** | .04 | – |  |  |  |  |  |  |  |
| 15. Sleep problems | 2.92 | 0.79 | .54** | .38** | .28** | -.35** | .55** | -.34** | .50** | -.21** | -.14** | .04 | -.12** | -.00 | .12* | -.10* | – |  |  |  |  |  |  |
| 16. Smartphone addiction | 3.13 | 1.06 | .34** | .33** | .26** | -.25** | .39** | -.25** | .40** | -.11* | -.01 | .00 | -.06 | -.08 | .08 | -.09* | .31** | – |  |  |  |  |  |
| 17. Videogames addiction | 1.97 | 1.03 | -.06 | .00 | -.06 | -.05 | -.07 | .01 | -.08 | -.02 | .00 | -.02 | .03 | .02 | -.08 | .07 | .01 | .24** | – |  |  |  |  |
| 18. Daytime spent with smartphone | 5.74 | 2.84 | .20** | .14** | .11** | -.16** | .21** | -.19** | .23** | -.08 | .01 | .01 | -.06 | .03 | .20** | -.03 | .27** | .35** | .15** | – |  |  |  |
| 19. Physical activity (n° hours) | 6.36 | 3.51 | -.05 | -.19** | -.10* | .02 | -.05 | .13** | -.07 | .08 | -.05 | -.06 | .01 | .06 | .01 | -.05 | -.02 | -.03 | -.01 | -.08 | – |  |  |
| 20. Sex assigned at birth | – | – | .32** | .26** | .25** | -.17** | .38** | -.25** | .38** | -.14** | .01 | .09* | -.10* | .00 | .13** | -.10* | .31** | .25** | -.32** | .14** | -.13** | – |  |
| 21. Age | 15.98 | 1.52 | .16** | .12** | .12** | -.07 | .12** | -.06 | .16** | -.10* | -.03 | .10* | -.12* | -.08 | .24** | -.11** | .16** | .00 | -.21** | .06 | -.07 | .10* | – |

*Note.* Sex assigned at birth (0 = *males*, 1 = *females*); **p* < .05, ***p* < .01.

**Table S2**

*Model Fit Indices for Each LPA Solution*

| Model | Log-likelihood | AIC | BIC | SABIC | Entropy | Smallest class % | LMR *p*-value | BLRT *p*-value |
| --- | --- | --- | --- | --- | --- | --- | --- | --- |
| 1-group | -5575.009 | 11178.019 | 11239.077 | 11194.633 | --- | --- | --- | --- |
| 2-groups | -5034.337 | 10112.675 | 10208.623 | 10138.782 | 0.81 | 47.3 | < .001 | < .001 |
| **3-groups** | **-4866.643** | **9793.285** | **9924.124** | **9828.886** | **0.82** | **19.5** | **< .001** | **< .001** |
| 4-groups | -4767.523 | 9611.046 | 9776.776 | 9656.141 | 0.80 | 16.6 | .01 | < .001 |
| 5-groups | -4722.119 | 9536.239 | 9736.859 | 9590.827 | 0.78 | 13.6 | .03 | < .001 |
| 6-groups | -4686.477 | 9480.954 | 9716.464 | 9545.035 | 0.79 | 5.0 | .41 | < .001 |

*Note.* In bold, the best LPA model solution. AIC = Akaike’s Information Criterion; BIC = Bayesian Information Criterion; SABIC = Sample-Adjusted BIC; LMR = Lo-Mendell Ruben; BLRT = Bootstrap Likelihood Ratio Test. In bold, we reported the best LPA solution.

**The Conditional LPA: The Three-Step Approach**

The three-step LPA approach was run on the 3-profile solution with the inclusion of 12 predictors. Results are reported in Table S3. The significant effects regard the variables (a) social support, (b) social support from relatives, (c) smartphone addiction, (d) sleep problems, and (e) physical activity. They exerted significant effects in establishing those who belong to high-risk subgroup vs (a) mild-risk and (b) low-risk subgroups. The significant and positive sign of the estimates indicates that an increase in each of those variables leads to an increased probability to belong to high-risk subgroup vs low-risk and mild-risk subgroups. In sum, those in the high-risk subgroup have higher values of sleep problems and smartphone addiction and lower values of physical activity compared to those in the low-risk and mild-risk subgroups. Results also indicated that the mild-risk subgroup reported lower values of social support in general and social support from relatives and higher scores of sleep problems and smartphone addiction compared to those in the low-risk subgroup.

**Table S3**

*Results from 3-step Procedure*

| Predictor | High-risk vs low-risk | | |  | High-risk vs mild-risk | | |  | Mild-risk vs low-risk | | |
| --- | --- | --- | --- | --- | --- | --- | --- | --- | --- | --- | --- |
|  | Estimate | LL 95%CI | UL 95%CI |  | Estimate | LL 95%CI | UL 95%CI |  | Estimate | LL 95%CI | UL 95%CI |
| Social support | -0.965 | -2.156 | 0.226 |  | -0.256 | -1.403 | 0.891 |  | **-0.709*** | **-1.291** | **-0.126** |
| Support from parents | -0.773 | -1.669 | 0.123 |  | -0.678 | -1.403 | 0.048 |  | -0.096 | -0.607 | 0.415 |
| Support from siblings | -0.620 | -1.381 | 0.141 |  | -0.468 | -1.169 | 0.233 |  | -0.153 | -0.541 | 0.236 |
| Support from relatives | -0.747 | -2.083 | 0.590 |  | 0.010 | -1.178 | 1.198 |  | **-0.756*** | **-1.424** | **-0.088** |
| Support from friends | 0.002 | -0.712 | 0.716 |  | -0.373 | -0.939 | 0.194 |  | 0.375 | -0.109 | 0.858 |
| Support from romantic partner | -0.368 | -0.941 | 0.204 |  | -0.175 | -0.714 | 0.365 |  | -0.194 | -0.498 | 0.111 |
| Support from teachers | -0.815 | -2.431 | 0.801 |  | -0.490 | -2.011 | 1.031 |  | -0.326 | -0.975 | 0.324 |
| Sleep problems | **3.650***** | **1.720** | **5.581** |  | **2.147**** | **0.513** | **3.780** |  | **1.504***** | **0.672** | **2.336** |
| Smartphone addiction | **1.874***** | **0.922** | **2.826** |  | **1.208**** | **0.418** | **1.999** |  | **0.666** | **0.177** | **1.155** |
| Video game addiction | -0.374 | -0.992 | 0.244 |  | -0.152 | -0.664 | 0.359 |  | -0.222 | -0.588 | 0.144 |
| Daytime spent with smartphone | 0.134 | -0.078 | 0.347 |  | 0.184 | -0.013 | 0.381 |  | -0.050 | -0.171 | 0.072 |
| Physical activity (n^o^ hours) | **-0.364**** | **-0.624** | **-0.104** |  | **-0.307**** | **-0.544** | **-0.069** |  | -0.057 | -0.152 | 0.038 |
|  | OR | LL 95%CI | UL 95%CI |  | OR | LL 95%CI | UL 95%CI |  | OR | LL 95%CI | UL 95%CI |
| Social support | 0.381 | 0.116 | 1.254 |  | 0.774 | 0.246 | 2.437 |  | **0.492** | **0.275** | **0.882** |
| Support from parents | 0.462 | 0.188 | 1.130 |  | 0.508 | 0.246 | 1.049 |  | 0.909 | 0.545 | 1.515 |
| Support from siblings | 0.538 | 0.251 | 1.151 |  | 0.626 | 0.311 | 1.263 |  | 0.858 | 0.582 | 1.266 |
| Support from relatives | 0.474 | 0.125 | 1.803 |  | 1.010 | 0.308 | 3.312 |  | **0.469** | **0.241** | **0.916** |
| Support from friends | 1.002 | 0.491 | 2.046 |  | 0.689 | 0.391 | 1.214 |  | 1.454 | 0.897 | 2.359 |
| Support from romantic partner | 0.692 | 0.390 | 1.226 |  | 0.840 | 0.489 | 1.441 |  | 0.824 | 0.608 | 1.117 |
| Support from teachers | 0.442 | 0.088 | 2.227 |  | 0.613 | 0.134 | 2.805 |  | 0.722 | 0.377 | 1.382 |
| Sleep problems | **38.492** | **5.585** | **265.292** |  | **8.556** | **1.671** | **43.808** |  | **4.499** | **1.958** | **10.339** |
| Smartphone addiction | **6.516** | **2.514** | **16.886** |  | **3.348** | **1.518** | **7.380** |  | **1.946** | **1.193** | **3.175** |
| Video game addiction | 0.688 | 0.371 | 1.415 |  | 0.859 | 0.515 | 1.433 |  | 0.801 | 0.555 | 1.074 |
| Daytime spent with smartphone | 1.144 | 0.925 | 1.277 |  | 1.202 | 0.987 | 1.464 |  | 0.952 | 0.843 | 1.155 |
| Physical activity (n^o^ hours) | **0.695** | **0.536** | **0.901** |  | **0.736** | **0.580** | **0.933** |  | 0.944 | 0.859 | 1.039 |

*Note.* A listwise deletion was applied to the auxiliary variables in the analysis (number of observations used *n* = 346), thereby, excluding a relevant portion of the total sample (*n* = 233). LL 95%CI = Lower Limit for 95% Confidence Interval; UL 95%CI = Upper Limit for 95% Confidence Interval; OR = Odds Ratio.

Estimates are unstandardized.

Bold indicates significant estimates; notice that to be significant the 95%CI should not include the value of 0 and 1 for "Estimate" and "OR", respectively.

^*^*p* < .05, ^**^*p* < .01, ^***^*p* < .001.
